# Copper release by MOF-74(Cu): a novel pharmacological alternative to diseases with deficiency of a vital oligoelement†

**DOI:** 10.1039/d3ra07109j

**Published:** 2024-01-02

**Authors:** Javier Aguila-Rosas, Betzabeth A. García-Martínez, Camilo Ríos, Araceli Diaz-Ruiz, Juan L. Obeso, Carlos T. Quirino-Barreda, Ilich A. Ibarra, Ariel Guzmán-Vargas, Enrique Lima

**Affiliations:** a Laboratorio de Fisicoquímica y Reactividad de Superficies (LaFReS), Instituto de Investigaciones en Materiales, Universidad Nacional Autónoma de México Circuito Exterior s/n, CU, Del. Coyoacán 04510 Ciudad de México Mexico aguzmanv@ipn.mx argel@unam.mx lima@iim.unam.mx; b Laboratorio de Farmacia Molecular y Liberación Controlada, Universidad Autónoma Metropolitana-Xochimilco Calzada del Hueso 1100, Col. Villa Quietud, C.P. 04960 CDMX Mexico; c Laboratorio de Neurofarmacología Molecular, Universidad Autónoma Metropolitana-Xochimilco Calzada del Hueso 1100, Col. Villa Quietud, C.P. 04960 CDMX Mexico; d Neurociencias Básica, Instituto Nacional de Rehabilitación Calz. México Xochimilco 289, Col. Arenal de Guadalupe, C.P. 14389 CDMX Mexico; e Dirección de Investigación, Instituto Nacional de Neurología y Neurocirugía Manuel Velasco Suárez Insurgentes Sur 3877, La Fama, Tlalpan CP14269 CDMX Mexico; f Laboratorio Nacional de Ciencia, Tecnología y Gestión Integrada del Agua (LNAgua), Instituto Politécnico Nacional, CICATA U. Legaria Legaria 694 Irrigación, Miguel Hidalgo CDMX Mexico; g Laboratorio de Investigación en Materiales Porosos, Catálisis Ambiental y Química Fina, Instituto Politécnico Nacional, ESIQIE-SEPI-DIQI UPALM Edif. 7 P.B. Zacatenco, GAM 07738 CDMX Mexico

## Abstract

Copper deficiency can trigger various diseases such as Amyotrophic Lateral Sclerosis (ALS), Parkinson's disease (PD) and even compromise the development of living beings, as manifested in Menkes disease (MS). Thus, the regulated administration (controlled release) of copper represents an alternative to reduce neuronal deterioration and prevent disease progression. Therefore, we present, to the best of our knowledge, the first experimental *in vitro* investigation for the kinetics of copper release from MOF-74(Cu) and its distribution *in vivo* after oral administration in male Wistar rats. Taking advantage of the abundance and high periodicity of copper within the crystalline-nanostructured metal–organic framework material (MOF-74(Cu)), it was possible to control the release of copper due to the partial degradation of the material. Thus, we simultaneously corroborated a low accumulation of copper in the liver (the main detoxification organ) and a slight increase of copper in the brain (striatum and midbrain), demonstrating that MOF-74(Cu) is a promising pharmacological alternative (controlled copper source) to these diseases.

## Abstract 2

Copper deficiency can trigger various diseases such as Amyotrophic Lateral Sclerosis (ALS), Parkinson's disease (PD) and even compromise the development of living beings, as manifested in Menkes disease (MS).

## Graphical abstract

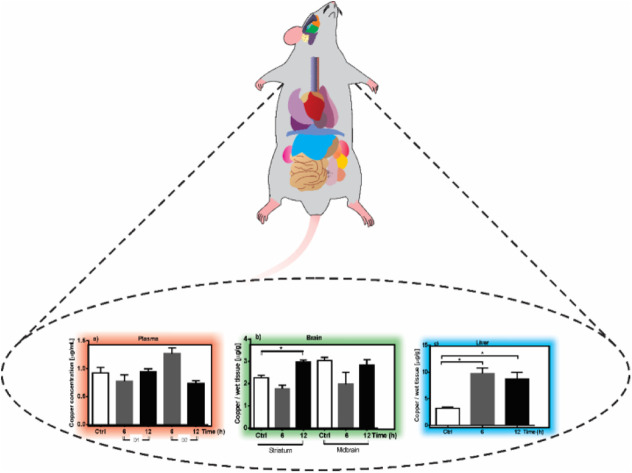

## Introduction

Worldwide, Parkinson's disease (PD) is the second most common neurodegenerative disease^1^ and is more common in men than women.^2^ Amyotrophic lateral sclerosis (ALS) is a poorly treated multifactorial neurodegenerative disease associated with multiple cell types and subcellular organelles. As with other multifactorial diseases, medications will likely need to target multiple disease processes.^3^ On the other hand, Menkes disease is a lethal, neurodegenerative, sex-linked hereditary childhood disease. This is considered an orphan disease.^4^

Developing therapeutic interventions for the central nervous system is challenging since therapy for these conditions focuses on addressing the symptomatology of these diseases and does not stop their progress.^5,6^ On the other hand, the first line of available treatments could generate side effects like the symptoms of the disease, and not all patients respond to a particular therapy with the same clinical diagnosis.^7^ Low concentrations of copper in the brain have been associated with Parkinson's disease^8^ and multiple sclerosis.^9^ Menkes syndrome is caused by a defect in the ATP7A gene. The defect makes it difficult for the body to properly distribute copper throughout the body. As a result, the brain and other parts of the body do not receive enough copper and it accumulates in the small intestine and kidneys. Therefore, the supply of this metal is recommended as an alternative to reduce neuronal deterioration and prevent disease progression.^10,11^ Cooper shows a vital biological relevance in human cells since it is an essential micronutrient in different human organs that perform a high metabolic activity, such as the liver, brain, kidneys, and heart.^12,13^ But this trace element also has an impact on the appearance and/or progression of Alzheimer's disease. Therefore, supplements containing high levels of inorganic copper, mainly in the form of inorganic copper sulfate (2 mg of inorganic copper per 500 mg tablet) cause manifestations of long-term hepatic/neurological/cognitive impairment in this condition.^14^ During oral administration of copper, the percentage of absorption at the intestinal level is dose and time dependent. Maintaining a concentration of 10 μM of copper at the extracellular level does not cause a first-pass effect. However, at higher concentrations presented by metal supplementation they promote a first-pass effect and consequently its elimination through bile sales until physiological balance is achieved.^15^

Copper is a critical functional cofactor of various enzymes, called cuproenzymes or metalloenzymes, which are essential in the physiological processes of living beings. For example, some of the vital functions of cooper in our organism are associated with energy production, connective tissue formation, iron metabolism, synthesis of chemical substances that are components of the central nervous system (noradrenaline and myelin) and oxidative processes.^16^

Metallothionein are a group of proteins made up of four isoforms present in a basal state of copper, however, isoform I and II are overexpressed by intracellular copper from supplementation with the aim of storing the metal and indirectly reducing the risk of cellular damage.^15^ However, Cu(ii), at relatively high concentrations, can exhibit toxicity since this can be accumulated in vital organs when this is inappropriately administered (high concentrations) and in a not gradual manner (low concentrations).^16^ For example, when our body is exposed to high Cu(ii) concentrations (*i.e.*, chronic exposure to copper), the liver is the main vital organ affected because this is the first deposit of this metal.^17^ Copper toxicity in the liver is mainly expressed as liver cirrhosis,^18^ which can gradually progress to severe adverse effects such as coma, liver necrosis, vascular collapse, and even death.^19^ Intoxication with Cu(ii) can cause weakness, lethargy and anorexia, gastrointestinal erosion, hepatocellular and kidney necrosis.^20^

Thus, advances in the application of innovative systems for the delivery of Cu(ii) could provide new strategies with the potential to significantly improve the treatment of Parkinson's disease.^21,22^ Therefore, some materials have been used in regenerative therapies to treat diseases at the central nervous system level.^6^ In recent years, selected metal–organic frameworks (MOFs) have been extensively investigated for the delivery of biological molecules and metals due to their easy functionalization, good biodegradability, and biocompatibility.^23,24^ MOF-74 is a well know family of MOFs with a wide variety of divalent metal cations coordinated to the 2,5-dioxide terephthalate (DOT) ligand,^25^ and it can be synthesized under green conditions with low-toxicity solvents.^26^ MOF-74(Cu) is typically identified for showing open metallic sites,^27^ a highly desirable characteristic for heterogeneous catalysis applications.^28^ However, its low chemical stability towards water significantly limits any plausible industrial application for this Cu(ii)-based MOF material.^29,30^

Such apparent disadvantage has been recently explored in potential clinical applications, since the degradation of MOF-74(Mg), under physiological conditions, gradually released Mg(ii) metal centers.^31,32^ MOF-74 (DOT) ligand was also reported as a non-toxic compound.^33^ This work inspired us to investigate the gradual decomposition of MOF-74(Cu) under a physiological environment. Recently, Angkawijaya and co-workers presented a comprehensive investigation on a biocompatible and biodegradable Cu(ii)-MOF based for tuberculosis treatment.^34^ Thus, with these prominent concepts in mind, we decided to investigate using MOF-74(Cu) as a novel and promising pharmacological alternative to treat diseases with deficiency of this metal.

## Experimental

Further details related to instrumental techniques are presented in the ESI.†

## Results and discussions

### Copper contained in MOF

To confirm the total copper content in the structure, acid digestion was performed, and the AA quantified it. The results shown in Table 1 confirm that the amount of copper in MOF-74(Cu) is 29.80% by weight metal/MOF.

**Table 1:** Copper concentration total

Sample	Copper (%)
MOF-74(Cu)	29.80

### *In vitro* studies of copper release

**Fig. 1:**
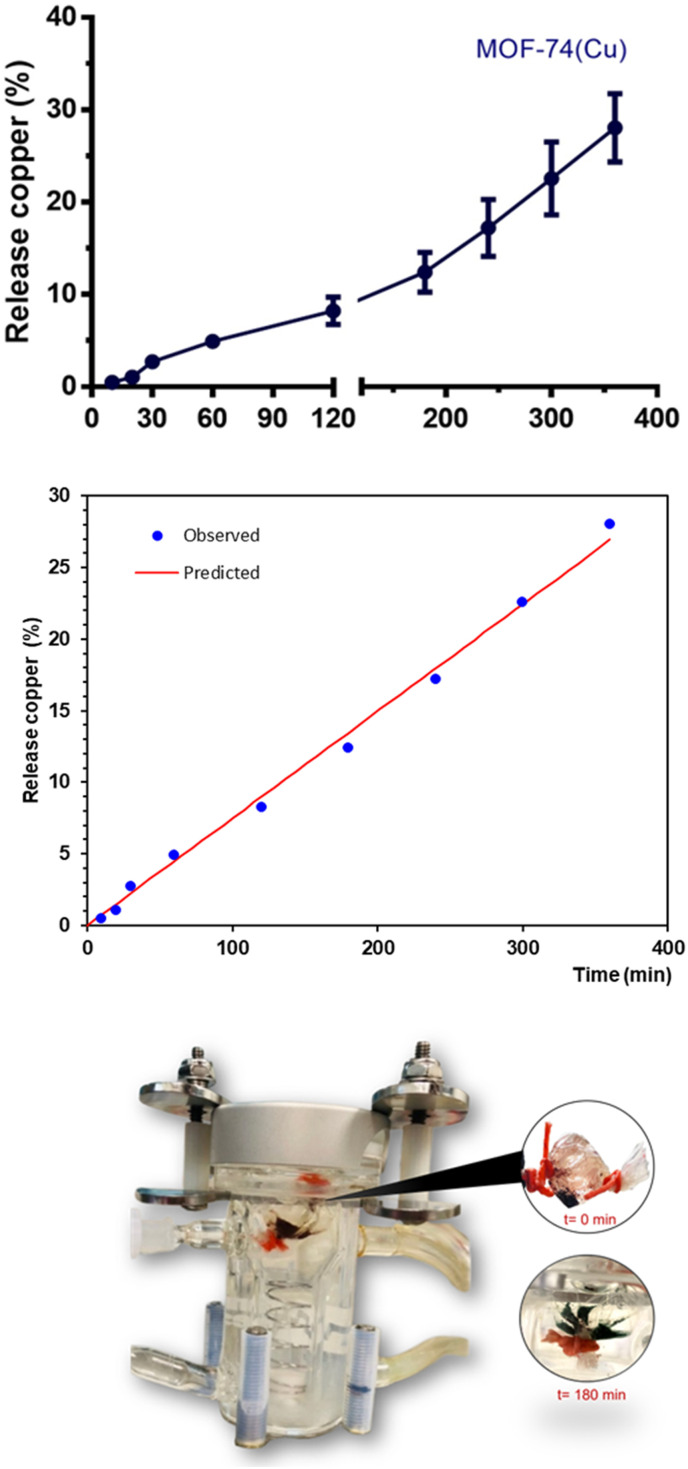
Top: Cu(ii) release profile from MOF-74(Cu). First section: 10 to 120 min. Second section: 120 to 360 min. Each bar represents the mean ± SD of *n* = 4 profiles by evaluated time. Middle: Release profile compared to zero release kinetics. Bottom: qualitative observation of MOF-74(Cu) degradation at 180 min.

The kinetics of Cu(ii) release from MOF-74(Cu) were evaluated *in vitro* using the dialysis membrane diffusion technique. The *in vitro* Cu(ii) release profiles from MOF-74(Cu) were evaluated in phosphate buffer with a physiological pH of pH 1, 2 for 10 minutes,^35^ to simulate the gastric emptying (process by which the contents of the stomach are moved into the duodenum) of the rat and subsequently changed to a solution of pH 7.4 similar to the conditions of the intestine. Fig. 1 shows that the average cumulative amount of Cu(ii) released is a growth curve. MOF-74(Cu) shows a low percentage of metal release and only reaches approximately 9% at 120 min, and it corresponds to the penetration of free metal through the membrane. The release was constant in the next period of 120 to 360 min. Therefore, approximately 10 to 30% of Cu(ii) is released from MOF-74(Cu).

Thus, a degradation of the MOF-74(Cu) structure, initiated at the experimental pH conditions, can be proposed. Certainly, this phenomenon can be qualitatively observed by showing a colour change from a characteristic red, for MOF-74(Cu), to a bright green characteristic of free Cu(ii) ions. So, we can propose that Cu(ii) release takes place under simulating gastrointestinal conditions, due to the partial degradation of MOF-74(Cu) (Fig. 1).^30,35^ After 180 min, after partial decomposition of MOF-74(Cu), the Cu(ii) release process is constant (Fig. 1, second section).

Release profile data were fitted to mathematical models to understand the kinetics of Cu(ii) release from MOF-74(Cu); thus, this could explain the possible release mechanisms involved. Six models, including zero order, first order, Higuchi, Hixson-Crowell, Korsmeyer-Peppas, and Weibull, were applied to the data set. The coefficient of determination (*R*^2^) value was used for fitting evaluation. The data is summarized in Table 2.

**Table 2:** Kinetic data for release cooper from MOF-74(Cu)

Zero-order	First-order	Higuchi	Korsmeyer-Peppas^a^	Hixson-Crowell	Weibull^b^
0.9947	0.9872	0.8352	0.9962, *n* = 1.089	0.9901	0.9958, *β* = 1.422

a *n* is the diffusion exponent in the Peppas-Korsmeyer model.

b *β* is the shape factor in the Weibull model.

The Cu(ii) release data from MOF-74(Cu) was best fitted to the zero-order kinetic model. In this model, the release is independent of the metal concentration. However, we can see that it also fits the Weibull kinetic model, which follows the eqn (1):1
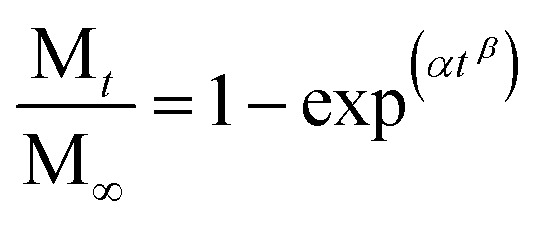
where M_*t*_/M_∞_ is a fraction of the metal released at time *t*, *α* defines the time scale of the process, and *β* characterizes the shape of the curve. The Weibull model is an empirical model that describes immediate and prolonged releases. The value of the exponent *b* is an important indicator related to the release mechanism of the metal through the system. Based on this value, it is possible to determine that the release of copper from MOF-74(Cu) is mediated by non-Fickian diffusion, and this is due to the surface degradation of MOF-74(Cu).^36^ In addition, this is confirmed by the value of “*n*” from the Korsmeyer-Peppas kinetic model obtained for our data, which indicates a value greater than 1 and corresponds to a non-Fickian diffusion.^37^

It is worth mentioning that the mechanism of copper absorption into the cell is achieved through a reduction process by the protein reductase, which reduces Cu^2+^ to Cu^+^ and facilitates diffusion through the Ctr1 transporter. Thus, although the metal supplied by MOF-74(Cu) is Cu(ii), Cu(i) is also present in different tissues. This work quantifies oxidation states Cu(ii) and Cu(i) in the evaluated tissues.

### Copper distribution: acute oral administration

For the study of the biodistribution of MOF-74(Cu), *in vivo* experiments were carried out in male Wistar rats. Considering the total copper content, the total mass of MOF-74(Cu) necessary to administer D1 (39.75 mg kg^−1^) and D2 (79.5 mg kg^−1^) of Cu(ii) was calculated. These doses were proposed based on previous research studies reported by our group,^38^ in which we evaluated the biodistribution of copper from an acute systemic administration of organic salt. We determined that a dose of 312 mg kg^−1^ and 156 mg kg^−1^ per body weight of the rat caused high toxicity and weight loss as a homeostasis uncontrol factor,^39^ respectively. A dose of 79.5 mg kg^−1^ body weight did not show any of these two harmful effects.

**Fig. 2:**
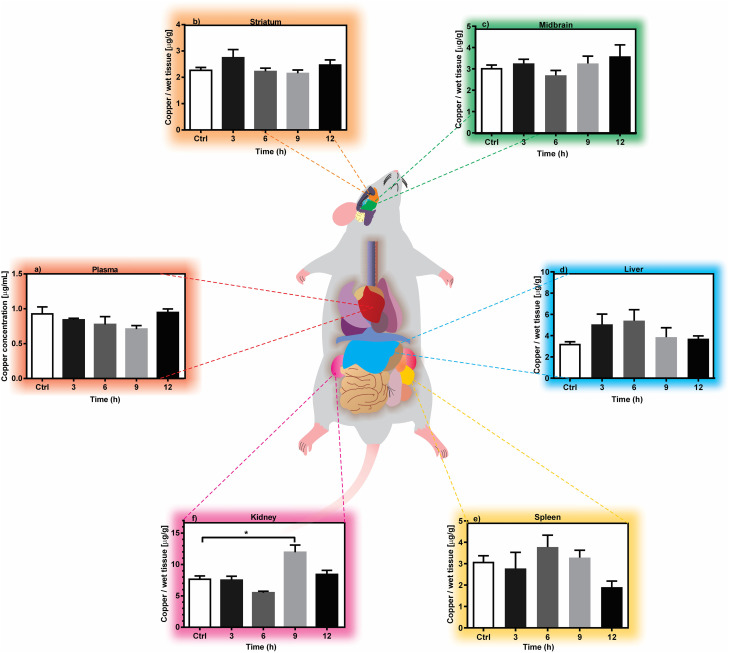
Time-course of Cu(ii) concentration in (a) plasma, (b) striatum, (c) midbrain, (d) liver, (e) kidney and (f) spleen tissue after oral administration of 39.75 mg Cu per kg. Each bar represents the mean ± SEM of *n* = 3–4 rats per group. A value of *p < 0.05 is a statistically significant difference in the means of copper concentration in each tissue analysed. The data were analysed using (a), (b) and (e) ANOVA test *vs.* control group (*t* = 0); (c), (d) and (f) Kruskal–Walli's test *vs.* control group (*t* = 0).

**Fig. 3:**
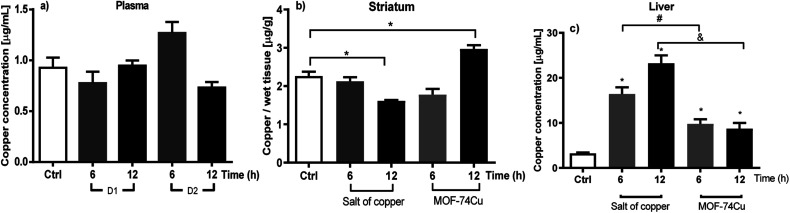
Comparison of the biodistribution of copper after acute oral administration of 79.5 mg per Cu kg in tissues such as (a) plasma, (b) striatum: copper gluconate and MOF-74(Cu) and (c) liver: copper gluconate and MOF-74(Cu). Each bar represents the mean ± SEM of *n* = 3–4 rats per group. *A value of *p* < 0.05 is a statistically significant difference in the means of copper concentration in each tissue analysed. The data were analysed using (a) and (b) ANOVA test *vs.* control group (*t* = 0); (c) Kruskal–Walli's test followed by Mann–Whitney-U tests *vs.* control group (*t* = 0). The results obtained from the distribution of copper as gluconate salt were gotten from García-Martínez *et al.* (2021),^38^ for comparison purposes only.

Therefore, this study proposes a first evaluation of only 50% of the reported dose (D2) as the non-copper saturated window in an *in vivo* study when evaluating a MOF-type carrier. Fig. 2 shows the monitoring of the copper concentration in plasma, brain (striatum and midbrain), liver, spleen and kidney at the different times evaluated (3, 6, 9 and 12 h) after oral administration for D1. Fig. 3 shows the monitoring of the concentration of copper in plasma, brain (striatum and midbrain) and liver for D2.

### Copper distribution in plasma

Plasma, as the first tissue analysed, is shown in Fig. 2a and 3a after the administration of D1 and D2, respectively. The found copper concentrations were not present a statistically significant variation over time for the control group. It has been reported that this phenomenon is common due to the high biodistribution of this metal at the systemic level.^40^ However, it has also been reported that if more sampling times beyond 12 hours are scheduled, plasma concentrations can be increased in this type of acute administration.^38^

### Copper distribution in the brain: striatum and midbrain

Since the tissues involved in Parkinson's disease, amyotrophic multiple sclerosis and Menkes disease are neurological tissues (striatum (Fig. 2b) and midbrain (Fig. 2c)), which present a decrease in copper and therefore.^43^ The compartmentalization of copper in these two brain regions was therefore monitored.^9,41^

The data did not show a statistically significant accumulation in both regions for D1. A slight increase in copper concentration can provide interesting results in an animal model miming the decrease in copper concentration for Parkinson's disease.^42^ For example; It has been reported in previous trials in murine models that increases in copper concentration in a range of 7.0 to 17.5% in the brain present a preventive effect against the damage generated during the mimicry of Parkinson's disease, such as lipoperoxidation of neuronal membranes, decrease in the concentration of dopamine in the substantia nigra pars compacta, the decrease in ferroxidase activity carried out by ceruloplasmin as well as neuronal apoptosis.^11,44^ But through a controlled release, since it has been reported that the abrupt accumulation of the metal can promote and participate in the production of reactive oxygen species and neuronal death, being another factor in the development of Alzheimer's disease.^45^

While the copper concentrations in this organ, after the administration of D2 (Fig. 3b), show statistically significant figures, with an increase of 31% for striated tissue at 12 h in comparison to the control group, in the midbrain the quantified Cu(ii) concentration continues without showing any increase or decrease when compared concerning the concentration of control group. This behavior is similar to relevant investigations previously reported in 2013 by intraperitoneal administration of copper sulfate (inorganic salt)^11^ and in 2020 through the administration of copper nanoparticles.^46^

### Distribution of copper in liver tissue

The liver is a key organ for monitoring the detoxification of copper.^47,48^ In this compartment, the accumulation of copper can rapidly occur to the point of full intoxication.^49^Fig. 2d shows the variation of the copper concentration as a function of time for D1.

The results do not show a statistically significant increase compared to the observed concentrations in the control group. This is beneficial because at 3, 9 and 12 h, only a slight increase in the concentration obtained is observed. This is possibly due to the overexpression of metallothioneins (MT; proteins responsible for metal retention) induced by the concentration of bioavailable metal after the administration of MOF-74(Cu).^50^

The degree of copper accumulation in this organ is related to the particle size,^40^ nature and shape of the carrier system (Paterson) and type of salt administered. It has been observed that Cu(ii) inorganic salts show low bioavailability and high absorption variability. Conversely, Cu(ii) organic salts exhibit greater absorption controlled by two absorption mechanisms: CTR1 transporters and passive diffusion.^51^

Our main motivation for choosing this MOF material as an alternative to supplying copper is related to its micrometric size (1.8 microns). It has been previously demonstrated that copper nanoparticles dissolve quickly, at relatively low pH, and this easily dissociates. Then, ionic particles in the stomach can cause high toxicity and copper accumulation in vital organs such as the liver. Thus, these complications are avoided when copper is introduced into an organism at the micron level (micro-sized particles).^40^

Remarkably, MOF-74(Cu) allows us to modify the gradual release of Cu(ii) in the stomach and intestine after 3 h (Fig. 1, section a) to be transported by CTR1. In addition, MOF-74(Cu) increases the bioavailability by passive diffusion since Cu(ii) continues as a coordinated-metal center in the rest of the MOF material (Fig. 1, section b).

Later, on administering D2 (Fig. 3c), it is possible to observe a significant accumulation of copper. This copper concentration (D2) increase was double-fold higher than the concentration in the control group at 12 hours (see Fig. 3c). However, the amount accumulated after the administration of D2 is still lower compared to our previous results, where it is observed that at the same administered dose of Cu(ii) gluconate, it shows an accumulation four-fold higher than the basal value 12 hours.^51^ The reduction of copper accumulation in the liver, as demonstrated by MOF-74(Cu), can reduce the production of free radicals and, therefore, decrease toxicity in the liver. The results obtained with D2 suggest that although a MOF system administers copper, the accumulation of copper is dose-dependent.

### Copper distribution in spleen and kidney

The spleen is an important immunological organ in the body that helps to defend the host (rat) and functions as a blood filter.^52^ In this study, it is considered a reference for monitoring the biodistribution of systemic copper since it has been observed retention (in the spleen) of different drug carrier systems (*e.g.*, carbon nanotubes), as well as metal nanoparticles.^40,53^Fig. 2e shows a copper accumulation of 22.2% at 6 h after D1 administration. It has been observed that this organ retains complex active molecules and metallic nanoparticles because these are larger than a free molecule. Therefore, they are recognized as antigenic particles.^54^

However, a decrease in copper concentration is observed at 9 and 12 h in the spleen. We could suggest that this is attributed to renal clearance (Fig. 2f) of the metal delivery system as a consequence of the presence of polar groups such as hydroxyl and carboxyl groups which constitute the ligand of MOF-74(Cu).^55^

Therefore, it is proposed that copper can be found as MOF-74(Cu) or as chelated metal fragments since it has been reported that the renal elimination pathway of the metal is not important after low-dose administration (up to 100 mg kg^−1^),^40^ being the main mechanism of copper excretion the bile pathway. On the other hand, there is no statistical significance in copper concentration in the spleen and liver tissues after the administration of D2 (Fig. S5†).

## Conclusions

In summary, we present, to the best of our knowledge, the first experimental *in vitro* study for the kinetics of copper-controlled release from MOF-74(Cu) and its distribution *in vivo* after oral administration in male Wistar rats. Remarkably, we observed a low accumulation of copper in the liver and a slight increase in its concentration in key tissues such as the striatum and midbrain, which is highly relevant in treating Parkinson's disease. It is worth to emphasize that the combination of a low concentration of copper in the liver while there is a small increase of copper concentration in the brain, are the ideal situation of copper-controlled release to alternative treat diseases with deficiency of this metal, such as Parkinson's disease, Amyotrophic Multiple Sclerosis and Menkes disease.

The crystalline structure of MOF-74(Cu) provides a suitable availability and high periodicity of copper within the MOF material, as well as its particle size, which corresponds to the micron scale, and the characteristic gradual decomposition of this copper-based MOF at a physiological pH (7.4) allowed us to achieve a controlled release like zero-order kinetics. Interestingly, these kinetics corroborated a slow and controlled release of copper, which represents low toxicity for the liver. Thus, this is huge step forwards over typically fast copper-release systems where a high accumulation of copper is commonly found in the liver.

Thus, our *in vivo* study where the compartmentalization of copper in different relevant tissues was demonstrated, representing an interesting and novel pharmacological alternative to Parkinson's disease, Amyotrophic Multiple Sclerosis and Menkes disease, since the risk of accumulation and intoxication by copper in the main detoxification organ, the liver, is considerably reduced while a slight increase of copper in the brain is required.

Finally, the corresponding results from the administration of D2 showed a significant change in copper concentrations at the brain level without compromising the concentrations of copper in the liver. Coupled with simple synthetic conditions (only methanol as a solvent and room temperature) and proven synthetic scalability, we believe that MOF-74(Cu) shows a strong promise for a pharmacological alternative, as a controlled copper source, to Parkinson's disease, Amyotrophic Multiple Sclerosis and Menkes disease.

## Ethical statement

All procedures with animals were carried out in accordance with the Guidelines for the care and use of laboratory animals from the LABORATORY ANIMAL PRODUCTION AND EXPERIMENTATION UNIT (UPEAL-BIOTERIO) of the University Autonomous Metropolitan and were approved by the Committee of Animal Ethics called: Internal Committee for the Care and Use of Laboratory Animals (CICUAL-UAM-X). Protocol number 170 called: EVALUATION OF PHARMACOTHERAPY WITH COPPER SALTS IN AN OPTIMAL PHARMACEUTICAL FORM IN AN EXPERIMENTAL MODEL OF PARKINSON'S DISEASE with opinion date 01/18/2017 and expiration date: 01/18/2025.

## Conflicts of interest

There are no conflicts to declare.

## Supplementary Material

RA-014-D3RA07109J-s001

## References

[cit1] Dorsey E. R., Elbaz A., Nichols E., Abd-Allah F., Abdelalim A., Adsuar C. J. (2018). Lancet Neurol..

[cit2] Nordengen K., Cappelletti C., Bahrami S., Frei O., Pihlstrøm L., Henriksen S. P., Geut H., Rozemuller A. J. M., van de Berg W. D. J., Andreassen O. A., Toft M. (2023). Brain: J. Neurol..

[cit3] Richardson P. J., Smith D. P., de Giorgio A., Snetkov X., Almond-Thynne J., Cronin S., Mead R. J., McDermott C. J., Shaw P. J. (2023). Transl. Neurodegener..

[cit4] e Vairo F. P., Chwal B. C., Perini S., Ferreira M. A. P., de Freitas Lopes A. C., Saute J. A. M. (2019). Mol. Genet. Metab..

[cit5] Cabreira V., Massano J. (2019). Acta Med. Port..

[cit6] Tam R. Y., Fuehrmann T., Mitrousis N., Shoichet M. S. (2014). Neuropsychopharmacol.

[cit7] Macher J.-P., Crocq M.-A. (2004). Clin. Neurosci..

[cit8] Bisaglia M., Bubacco L. (2020). Biomolecules.

[cit9] Roberts B. R., Lim N. K. H., McAllum E. J., Donnelly P. S., Hare D. J., Doble P. A., Turner B. J., Price K. A., Lim S. C., Paterson B. M., Hickey J. L., Rhoads T. W., Williams J. R., Kanninen K. M., Hung L. W., Liddell J. R., Grubman A., Monty J. F., Llanos R. M., Kramer D. R., Mercer J. F. B., Bush A. I., Masters C. L., Duce J. A., Li Q. X., Beckman J. S., Barnham K. J., White A. R., Crouch P. J. (2014). J. Neurosci..

[cit10] Gromadzka G., Tarnacka B., Flaga A., Adamczyk A. (2020). Int. J. Mol. Sci..

[cit11] Rubio-Osornio M., Montes S., Heras-Romero Y., Guevara J., Rubio C., Aguilera P., Rivera-Mancia S., Floriano-Sánchez E., Monroy-Noyola A., Ríos C. (2013). Neurosci. Res..

[cit12] Chen X., Liu J., Li Y., Pandey N. K., Chen T., Wang L., Amador E. H., Chen W., Liu F., Xiao E. (2022). Bioact. Mater..

[cit13] Ackerman C. M., Chang C. J. (2018). J. Biol. Chem..

[cit14] Squitti R., Catalli C., Gigante L., Marianetti M., Rosari M., Mariani S., Rongioletti M. (2023). Int. J. Mol. Sci..

[cit15] Linder M. C. (2020). Int. J. Mol. Sci..

[cit16] Montes S., Rivera-Mancia S., Diaz-Ruiz A., Tristan-Lopez L., Rios C. (2014). Oxid. Med. Cell. Longevity.

[cit17] Wang B., Wang X.-P. (2019). Curr. Neuropharmacol..

[cit18] Liu H., Guo H., Jian Z., Cui H., Fang J., Zuo Z., Deng J., Li Y., Wang X., Zhao L. (2020). Oxid. Med. Cell. Longevity.

[cit19] Kahlson M. A., Dixon S. J. (2022). Science.

[cit20] Wang K., Ma J.-Y., Li M.-Y., Qin Y.-S., Bao X.-C., Wang C.-C., Cui D.-L., Xiang P., Ma L. Q. (2021). Sci. Total Environ..

[cit21] Mittal K. R., Pharasi N., Sarna B., Singh M., Rachana R., Haider S., Singh S. K., Dua K., Jha S. K., Dey A., Ojha S., Mani S., Jha N. K. (2022). Transl. Neurosci..

[cit22] Kiran Raj G., Singh E., Hani U., Ramesh K. V. R. N. S., Talath S., Garg A., Savadatti K., Bhatt T., Madhuchandra K., Osmani R. A. M. (2023). J. Controlled Release.

[cit23] Zong Z., Tian G., Wang J., Fan C., Yang F., Guo F. (2022). Pharmaceutics.

[cit24] Medel E., Obeso J. L., Serrano-Fuentes C., Garza J., Ibarra I. A., Leyva C., Inge A. K., Martínez A., Vargas R. (2023). Chem. Commun..

[cit25] Kim H., Hong C. S. (2021). CrystEngComm.

[cit26] Flores J. G., Sánchez-González E., Gutiérrez-Alejandre A., Aguilar-Pliego J., Martínez A., Jurado-Vázquez T., Lima E., González-Zamora E., Díaz-García M., Sánchez-Sánchez M., Ibarra I. A. (2018). Dalton Trans..

[cit27] Kökçam-Demir Ü., Goldman A., Esrafili L., Gharib M., Morsali A., Weingart O., Janiak C. (2020). Chem. Soc. Rev..

[cit28] Choe J. H., Kim H., Hong C. S. (2021). Mater. Chem. Front..

[cit29] Voskanyan A. A., Goncharov V. G., Novendra N., Guo X., Navrotsky A. (2020). ACS Omega.

[cit30] Zuluaga S., Fuentes-Fernandez E. M. A., Tan K., Xu F., Li J., Chabal Y. J., Thonhauser T. (2016). J. Mater. Chem. A.

[cit31] Lawson S., Rownaghi A. A., Rezaei F. (2022). ACS Appl. Bio Mater..

[cit32] Ramos D., Aguila-Rosas J., Quirino-Barreda C. T., Santiago-Tellez A., Lara-García H. A., Guzmán A., Ibarra I. A., Lima E. (2022). J. Mater. Chem. B.

[cit33] Kuriyama I., Nakajima Y., Nishida H., Konishi T., Takeuchi T., Sugawara F., Yoshida H., Mizushina Y. (2013). Mol. Med. Rep..

[cit34] Angkawijaya A. E., Bundjaja V., Santoso S. P., Go A. W., Lin S.-P., Cheng K.-C., Soetaredjo F. E., Ismadji S. (2023). Biomater. Adv..

[cit35] Rosnes M. H., Pato-Doldán B., Johnsen R. E., Mundstock A., Caro J., Dietzel P. D. C. (2020). Microporous Mesoporous Mater..

[cit36] Dash S., Murthy P. N., Nath L., Chowdhury P. (2010). Acta Pol. Pharm..

[cit37] Geeva Prasanth A., Sathish Kumar A., Sai Shruthi B., Subramanian S. (2020). Mater. Res. Express.

[cit38] García-Martínez B. A., Montes S., Tristán-López L., Quintanar-Guerrero D., Melgoza L. M., Baron-Flores V., Ríos C. (2021). BioMetals.

[cit39] Adamson S. X.-F., Zheng W., Agim Z. S., Du S., Fleming S., Shannahan J., Cannon J. (2021). Biomolecules.

[cit40] Lee I.-C., Ko J.-W., Park S.-H., Shin N.-R., Shin I.-S., Moon C., Kim J.-H., Kim H.-C., Kim J.-C. (2016). Part. Fibre Toxicol..

[cit41] Waninger S., Berka C., Stevanovic Karic M., Korszen S., Mozley P. D., Henchcliffe C., Kang Y., Hesterman J., Mangoubi T., Verma A. (2020). J. Parkinson's Dis..

[cit42] Gou D.-H., Huang T.-T., Li W., Gao X.-D., Haikal C., Wang X.-H., Song D.-Y., Liang X., Zhu L., Tang Y., Ding C., Li J.-Y. (2021). Redox Biol..

[cit43] Davies K. M., Mercer J. F. B., Chen N., Double K. L. (2016). Clin. Sci..

[cit44] Islas-Cortez M., Rios C., Rubio-Osornio M., Zamudio S., Orozco-Suarez S., Mendez-Armenta M., Diaz-Ruiz A. (2021). Neurotoxicology.

[cit45] Dusek P., Roos P. M., Litwin T., Schneider S. A., Flaten T. P., Aaseth J. (2015). J. Trace Elem. Med. Biol..

[cit46] Fahmy H. M., Ali O. A., Hassan A. A., A Mohamed M. (2020). J. Trace Elem. Med. Biol..

[cit47] Luza S., Speisky H. (1996). Am. J. Clin. Nutr..

[cit48] Zatulovskaia Y. A., Ilyechova E. Y., Puchkova L. V. (2015). PLoS One.

[cit49] Kjærgaard K., Sandahl T. D., Frisch K., Vase K. H., Keiding S., Vilstrup H., Ott P., Gormsen L. C., Munk O. L. (2020). Radiopharm. Chem..

[cit50] Hauser-Davis R. A., Bastos F. F., Tuton B., Chávez Rocha R., Pierre T. S., Ziolli R. L., Arruda M. A. Z. (2014). J. Trace Elem. Med. Biol..

[cit51] Myint Z. W., Oo T. H., Thein K. Z., Tun A. M., Saeed H. (2018). Ann. Hematol..

[cit52] Lewis S. M., Williams A., Eisenbarth S. C. (2019). Sci. Immunol..

[cit53] Tang H., Xu M., Zhou X., Zhang Y., Zhao L., Ye G., Shi F., Lv C., Li Y. (2018). Mater. Sci. Eng., C.

[cit54] Cataldi M., Vigliotti C., Mosca T., Cammarota M., Capone D. (2017). Int. J. Mol. Sci..

[cit55] Baati T., Horcajada P., Gref R., Couvreur P., Serre C. (2011). J. Pharm. Biomed. Anal..

