# Supplementary material for: Copper release by MOF-74(Cu): a novel pharmacological alternative to diseases with deficiency of a vital oligoelement
Source: RSC Adv. 2024 Jan 2;14(2):855–62. doi: 10.1039/d3ra07109j (PMC10759266; doi:10.1039/d3ra07109j)
Supplement: RA-014-D3RA07109J-s001 [file RA-014-D3RA07109J-s001.pdf]

Electronic Supplementary Material (ESI) for RSC Advances.
This journal is © The Royal Society of Chemistry 2023

Supplementary Information for:

Copper release by MOF-74(Cu): a novel pharmacological alternative to diseases with deficiency of vital oligoelement

Javier Aguila-Rosas,[†a,b] Betzabeth A. García Martínez,[†c,d] Camilo Ríos,[c,d] Araceli Diaz-Ruiz,[e] Juan L. Obeso,[a] Carlos T. Quirino-Barreda[b] Ilich A. Ibarra[*,a]Ariel Guzmán-Vargas[g*]and Enrique Lima[*,a]

[a]Laboratorio de Fisicoquímica y Reactividad de Superficies (LaFReS), Instituto de Investigaciones en Materiales, Universidad Nacional Autónoma de México, Circuito Exterior s/n, CU, Del. Coyoacán, 04510, Ciudad de México, Mexico.

[b]Laboratorio de Farmacia Molecular y liberación controlada. Universidad Autónoma Metropolitana-Xochimilco. Calzada del Hueso 1100, Col. Villa Quietud, C.P. 04960, CDMX, México.

[c]Laboratorio de Neurofarmacología Molecular. Universidad Autónoma Metropolitana-Xochimilco. Calzada del Hueso 1100, Col. Villa Quietud, C.P. 04960, CDMX, México.

[d]Neurociencias Básica, Instituto Nacional de Rehabilitación. Calz. México Xochimilco 289, Col. Arenal de Guadalupe, C.P. 14389, CDMX, México.

[e]Dirección de investigación, Instituto Nacional de Neurología y Neurocirugía Manuel Velasco Suárez, Insurgentes Sur 3877, La Fama, Tlalpan, CP14269 CDMX, México.

[f]Instituto Politécnico Nacional, CICATA U. Legaria, Laboratorio Nacional de Ciencia, Tecnología y Gestión Integrada del Agua (LNAgua), Legaria 694 Irrigación, Miguel Hidalgo, CDMX, México.

[g]Instituto Politécnico Nacional, ESIQIE-SEPI-DIQI, Laboratorio de Investigación en Materiales Porosos, Catálisis Ambiental y Química Fina, UPALM Edif. 7 P.B. Zacatenco, GAM, 07738 CDMX, México.

[+]These authors contributed equally to this work.

Table of contents

S1. Experimental details..S3

S2. Results and Discussions..S5

S3. References...S7

S1. Experimental details

Materials

The following reagents were purchased from Sigma® USA and used without further purification: 2-Hydroxyterephthalic acid (H_2BDC-OH, 97%), Copper acetate monohydrate ($Cu(CO_2CH_3)_2 \cdot H_2O$, 98%), methanol ($CH_3OH$, 99%), and nitric acid (HNO_3 70%, purified by redistillation ≥ 99.999% trace metals basis). Deionized water and hydrochloric acid (HCl) was supplied by Milli-Q® and J.T. Baker®, respectively. Standard-grade regenerated cellulose membrane of dialysis was supplied by Spectrum™ Labs Spectra. Perkin Elmer's copper standard solution (1000 µg/mL) was obtained from Perkin Elmer Mexico.

MOF-74(Cu) synthesis

The sample MOF-74(Cu) was synthesised as follows: $Cu(CO_2CH_3)_2 \cdot H_2O$ (2 mmol, 0.40 g), H_2BDC-OH (17.5 mmol, 2.88g) were dissolved in methanol (104 mmol, 3.33g). The mixture was stirred until obtaining a homogeneous solution. After 20 h, the red powder was separated by centrifugation at 2500 rpm, and it was washed several times with methanol and then dried under vacuum at 100 °C for 12 h.

Characterization methods

X-ray diffraction (XRD) patterns were obtained using a D8 Advance (Bruker®), with a copper Kα radiation source. Thermal stability of samples was followed by Thermogravimetric Analysis (TGA) in equipment by TA Instruments®. The determination of particle size for MOF-74(Cu), 1.8 microns, was determined by dispersing a sample of the Cu(II)-MOF material in methanol and analyzed by dynamic light scattering (DLS) (Litesizer 500 Anton Paar®).

Release profiles

For in-vitro experiments, a known quantity of MOF-74(Cu) was immersed in 8 mL of preheated phosphate-buffered saline (PBS) solution (0.1M) at pH 1,2 for 10 minutes, to simulate the gastric emptying of the rat and subsequently changed to a solution of pH 7.4 similar to the conditions of the intestine. Vials were sealed andmaintained at 37°C±1°C and stirred. An aliquot of 100 µL was withdrawn at different times(10, 20, 30, 60, 120, 240, 300, and 360 min) and replaced with the same volume of fresh dissolutionmedium. The aliquots were filtered by a 0.2 µm pore size membrane.

Animals

Male Wistar rats weighing between 260 and 290 g (8.8 ± 0.35 weeks old) were used, and these were provided by the Animals Laboratory Production and Experimentation Unit (UPEAL for its Spanish acronym) from the Metropolitan Autonomous University-Xochimilco (UAM-X, Mexico). The animals were housed under standard laboratory conditions with a 12 h light/dark cycle and had ad-libitum access to food (Laboratory Rodent Diet 501) and water. All experimental procedures were approved by the Internal Committee for the Care and Use of Laboratory Animals from the same institution (Protocol No. 170). Additionally, the guidelines of the Official Mexican Standard (NOM-062-ZOO-1999) from the Secretariat of Agriculture, Livestock Rural Development, Fisheries and Food (1999) were followed. All tests were performed during daylight hours, and the number of experimental animals was kept at a minimum (n=3-4).

Oral administration of MOF-74(Cu)

A dispersion of MOF-74(Cu) equivalent to doses of 39.75(D1) or 79.5 mg/kg (D2) of Cu(II) was administered by oral gavage to different groups of rats. After that, the groups that received D1 were sacrificed by decapitation at 3, 6, 9, and 12 h post-treatment. While the groups treated with the higher dose (D2) were sacrificed at 6 and 12 h. Samples of the blood, striatum, midbrain, liver (left lateral lobe), kidney, and spleen were collected at each time. Plasma samples were obtained by blood centrifugation at 3500 rpm for 10 min and stored at − 21 °C until copper determination. The brain tissues were dissected on ice and immediately weighed. Animals in the control group received the vehicle (deionised water) and equimolar doses of H_2BDC-OH, (2,5-dioxide terephthalate, DOT), which were sacrificed immediately (zero time). The amount of copper found in the different tissues and blood samples of the control group was considered the baseline value. All samples were kept at -21 °C until analysis.

Copper quantification assay

Copper (I and II) determination was performed using an atomic absorption spectrophotometer (AA) (Perkin-Elmer® 3110) at a wavelength of 324.8 nm and equipped with an HGA-600 graphite furnace and an AS-60 autosampler. Copper concentrations were calculated using a six-point calibration curve over a copper concentration range of 2 to 45 μg/L copper, using the standard dilution method with 0.2% Nitric acid 65% Suprapur®. All the curves presented a coefficient of determination ≥ 0.99. The method was validated to demonstrate whether it complies with the identification and quantification of the metal, in a precise, exact, and robust manner.
Tissue samples: acid digestion of the different tissues was performed by adding 0.3 mL of supra pure HNO_3 and shaking the samples in a water bath at 60 °C for 30 min.
The total charge of Cu(II) in the MOF-74(Cu): 2 mg of MOF-74(Cu) were weighed in triplicate, and 5 mL of a 1M H_3PO_4 solution was added. The resultant mix was shacked in a water bath for 24 h at 50°C and 60 rpm. All samples were diluted in 0.2% v/v with supra pure HNO_3. Results were reported in micrograms of copper per gram of wet tissue (μg/g) fortissues and micrograms per milliliter (μg/mL) for plasma samples and the samples obtained from release profiles tests.

Statistical analysis

One-way analysis of variance (ANOVA) followed by a Dunnett´s test was used for data with normal distribution and variance homogeneity. For data that did not meet the requirements for parametric analysis results Kruskal–Wallis and Mann-Whitney-U tests were used, establishing statistical significance at *$p<0.05$ for all tests.

S2. Results and Discussions

MOF-74(Cu) characterization

Figure S1 shows the PXRD pattern of MOF-74(Cu). PXRD patterns display crystalline structures fitting to that of MOF-74(M) previously reported.[1] The TGA profile shown in Figure S2 confirms the low thermal stability of MOF-74(Cu). The sample presents a weight loss close to 23%, in the temperature range between 30 and 100°C, which is expected. In the following temperature range, the MOF-74(Cu) sample shows an additional loss of close to 5%. So that it finally degrades with greater weight loss from 234°C. In comparison, the mean particle size is shown in Table S1.

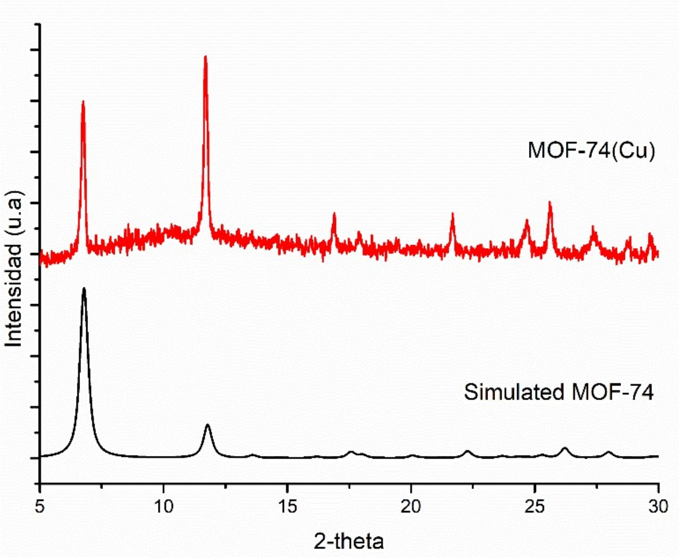

Figure S1. Experimental and simulated XRD patterns of the MOF-74(Cu).

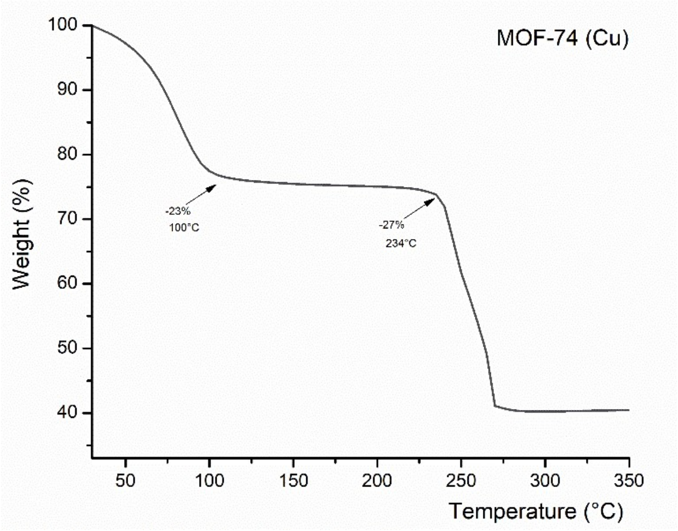

Figure S2. TGA pattern of MOF-74(Cu).

Table S1. Particle size

Sample	Mean (μm)	SD
MOF-74 (Cu)	1.801	0.1858

**The data shown is the result of 5 measurements for each sample.*

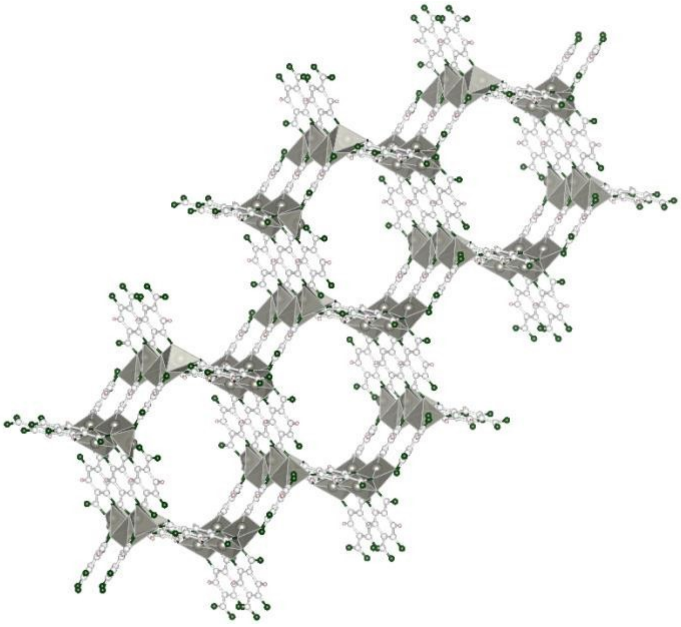

Figure S3. Structural molecule of MOF-74.

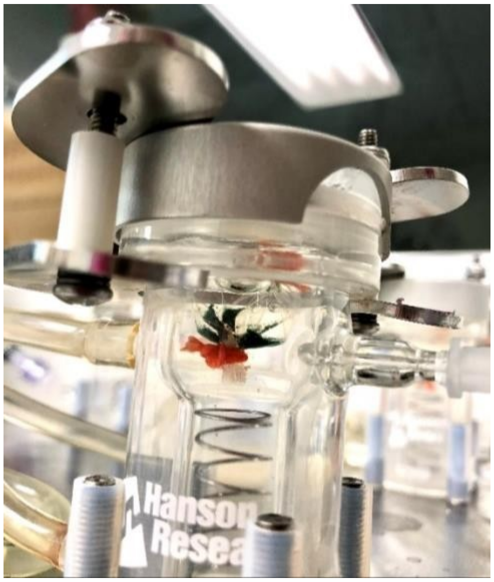

Figure S4. Pattern of MOF-74(Cu) after de 180 min.

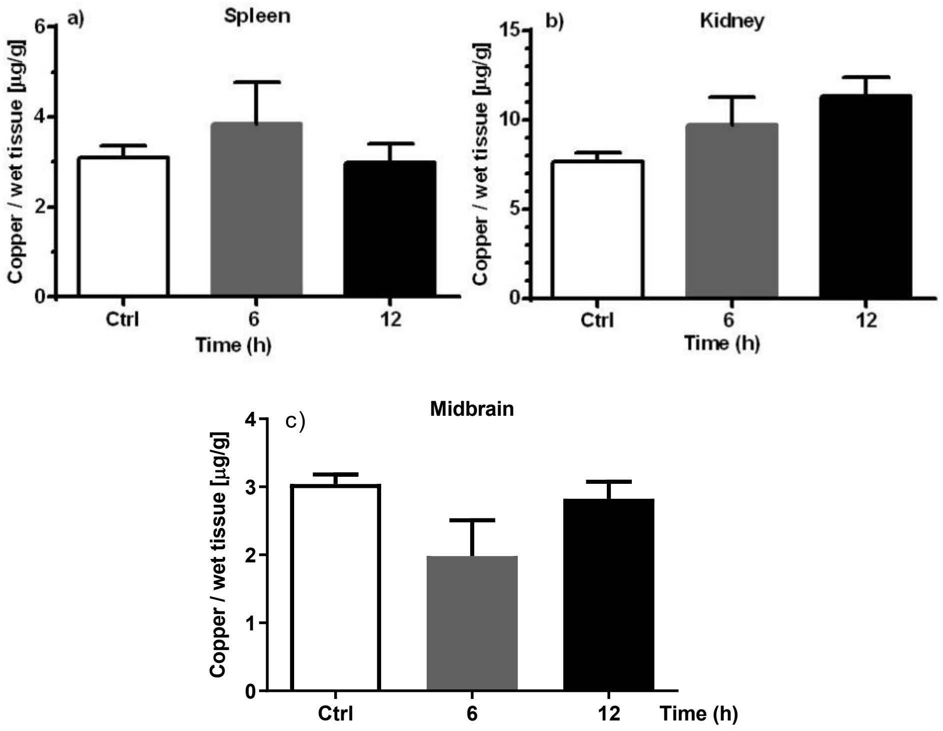

Figure S5. Comparison of the biodistribution of copper after acute oral administrationof 79.5 mg Cu/kg in tissues such as a) spleen, b) kidney and c) Midbrain. Each bar represents themean ± SEM of n = 3–4 rats per group. *A value of $p<0.05$ as a statistically significant difference in the means of copper concentration in each tissue analysed. The data were analysed using the ANOVA test vs the control group (t=0).

S3. References

[1] J. G. Flores, E. Sánchez-González, A. Gutiérrez-Alejandre, J. Aguilar-Pliego, A. Martínez, T. Jurado-Vázquez, E. Lima, E. González-Zamora, M. Díaz-García, M. Sánchez-Sánchez, I. A. Ibarra, *Dalt. Trans.* **2018**, *47*, 4639–4645.
